# The Long Path to Motherhood: Personal Growth and Well-Being During Medically Assisted Reproduction

**DOI:** 10.3390/bs15121694

**Published:** 2025-12-06

**Authors:** Elad Mijalevich-Soker, Roni Meir, Orit Taubman – Ben-Ari

**Affiliations:** 1 Psychology Department, Bar-Ilan University, Ramat Gan 5290002, Israel; elad.mijalevich@biu.ac.il; 2 School of Social Work, Bar-Ilan University, Ramat Gan 5290002, Israel; ronimeir93@gmail.com

**Keywords:** well-being, personal growth, medically assisted reproduction, infertility, self-mastery, emotional regulation

## Abstract

Most studies of medically assisted reproduction (MAR) primarily focus on its negative consequences for women’s mental health. However, there are indications that women experience well-being and personal growth during this period as well. This study focuses on personal and social resources that contribute to women’s well-being and personal growth during MAR, based on Schaefer and Moos’s personal growth model. We examined the contributions of socio-demographic and fertility-related characteristics, self-mastery, emotional regulation, and the tendency for social sharing. Women (*n* = 175) undergoing MAR completed self-report questionnaires. The study’s variables accounted for 36.6% of the variance in well-being and 34.4% in personal growth, indicating that higher self-mastery was associated with both higher well-being and personal growth. A lower tendency to suppress emotions was related to higher well-being, and a greater inclination for reappraisal and more sharing of the situation with others were associated with greater personal growth. Additionally, women undergoing in vitro fertilization (IVF) experienced more growth compared to other MAR procedures. The findings highlight the importance of self-mastery, reappraisal, and social sharing for well-being and personal growth during MAR. They indicate that these positive consequences are distinct constructs, that deserve different intervention strategies to empower women’s mental health in such circumstances.

## 1. Introduction

The formal definition of infertility is a failure to conceive through a year or more of unprotected heterosexual intercourse (Mascarenhas et al., 2012; World Health Organization, 2023). A recent global estimate of infertility prevalence indicates that 17.5% of individuals have experienced infertility at some point in their lives, and the estimated period prevalence (individuals/couples with infertility at a given point or interval in time) stands at 12.6% (Cox et al., 2022; World Health Organization, 2023). As a response to this medical problem, Medically Assisted Reproduction (MAR) procedures are offered, encompassing a variety of treatments aimed at facilitating conception for individuals suffering infertility, including ovulation induction, intrauterine insemination (IUI), and in vitro fertilization (IVF) (Goisis et al., 2023).

Receiving an infertility diagnosis often results in sorrow and harsh emotional reactions (Boivin et al., 2023), particularly for women, due to the prevailing perception in many cultures that they bear the primary responsibility for reproduction and family continuity (A. Greil et al., 2011; Luk & Loke, 2015). The mental health of women coping with fertility problems can vary as a function of their decisions concerning treatment (Shreffler et al., 2020).

Research indicates that women who opt for MAR often experience higher levels of distress compared to those who have been diagnosed with infertility but chose not to seek treatment (A. L. Greil et al., 2010, 2011). This seems to be due to the personal toll and physical demands of treatment, which can heighten the risk of suffering anxiety and depression (Kiani et al., 2021; Klitzman, 2018). Women undergoing MAR have to confront discomfort and pain stemming from medical procedures, complex relationships with fertility specialists, daily routine disruption, and uncertainty (Benyamini et al., 2005; Borghi et al., 2021). Furthermore, anticipating treatment results and waiting during and between treatment cycles constitute another dimension of distress and frustration (Boivin et al., 2012; Palomba et al., 2018).

### 1.1. Positive Outcomes During MAR

While the MAR process is mostly discussed in negative terms (Kiani et al., 2021; Rooney & Domar, 2018), it can also entail positive aspects such as well-being and personal growth (Abu-Sharkia et al., 2023; Paul et al., 2010; Yu et al., 2014). However, research into these aspects has been very limited. To fill this gap, the current study focused on positive aspects that women can experience during MAR. Specifically, we examined the contribution of socio-demographic and fertility-related characteristics, self-mastery, emotional regulation, and social sharing to women’s well-being and personal growth during MAR.

This study is based on Schaefer and Moos’s (1992, 1998) personal growth model, which suggests that event-related characteristics, coping resources, and environmental and internal resources can separately or collectively contribute to positive outcomes in challenging situations. Their model emphasizes the importance of considering several components from a more holistic perspective to explain what contributes to personal growth. This approach highlights the significance of social and sociodemographic elements, as well as the characteristics of the event and coping mechanisms, rather than concentrating only on personal resources. In this study, we will address two positive aspects, well-being and personal growth, thus extending the focus of Schaefer and Moos’s model to an additional positive outcome.

### 1.2. Well-Being and MAR

**Well-being** is a positive emotional state characterized by a feeling of happiness and peacefulness, as opposed to distress, which is characterized by a negative state accompanied by irritability and excessive worries (Stewart et al., 1988; Veit & Ware, 1983). Several variables were found to contribute to women’s well-being during MAR. Thus, women who tend to deal with their distress through meaning-based coping and who use fewer avoidance strategies reported higher quality of life (Swift et al., 2023); Better physical health was linked to greater life satisfaction and positive affectivity (Ben Shlomo et al., 2019); and women who reported that their mothers provided more active support during MAR (i.e., were more involved in their emotional state and attentive to their needs) showed higher levels of well-being, whereas those who experienced maternal overprotection (i.e., the provision of unnecessary help) experienced greater distress (Skvirsky et al., 2018).

### 1.3. Personal Growth and MAR

**Personal growth** (or post-traumatic growth, Tedeschi et al., 2018) is defined as the experience of positive changes in one’s life following coping with stressful circumstances (Schaefer & Moos, 1992; Tedeschi & Calhoun, 2004). More broadly, this positive transformation is manifested following a process in which individuals successfully rebuild their core beliefs that were disrupted by stressful circumstances or events (Cann et al., 2010). This reconstruction can be a byproduct of a combination of growth-related processes, such as cognitive (event meaning reframing), emotional (development of emotion regulation strategies), and social (support from others) (Tedeschi et al., 2018). For example, women may struggle to cope with a diagnosis of infertility for themselves or their spouse. This revelation can have a profound impact on the foundational aspects of a woman’s feminine identity and challenge her beliefs about future motherhood (Rooney & Domar, 2018), leading to personal growth.

We use the term “personal growth” following Schaefer and Moos (1992, 1998), rather than “post-traumatic growth” to avoid implying that the perinatal period or MAR constitute traumatic events. Instead, we conceptualize them as stress-related circumstances. This approach is consistent with Tedeschi et al. (2018), given that not all stressful events are necessarily traumatic, and given that growth is not only experienced following severe stress.

Among the few studies that examined personal growth in the context of MAR, one found that women who coped more flexibly with the challenges of MAR had lower levels of perceived stress, higher experiences of personal growth, and greater life satisfaction (Abu-Sharkia et al., 2023). Another study discovered that receiving more support from others during treatments was associated with greater resilience and experience of personal growth (Yu et al., 2014). Similarly, women’s greater satisfaction with their available social support was related to higher growth (Paul et al., 2010).

It is important to note that well-being and personal growth are distinct concepts (Ruini et al., 2015; Taubman – Ben-Ari, 2019; Tedeschi et al., 2018). Although they are correlated to some degree and share some common characteristics (Chan et al., 2016; Helgeson et al., 2006), their essence differs. Personal growth is examined in relation to a specific stressful event or condition, and denotes change (Tedeschi et al., 2018), whereas well-being is a more generalized sense of positive emotions (Stewart et al., 1988; Topp et al., 2015).

### 1.4. Contributors to Well-Being and Personal Growth During MAR

Two variables which might be related to positive aspects of coping with infertility are self-mastery and emotional regulation, which constitute psychological resources considered as significant contributors to women’s well-being and personal growth in other life circumstances. However, these variables have rarely been studied in the context of MAR.

**Self-mastery** refers to an individual’s perception of their control over life events (Pearlin & Schooler, 1978). Self-mastery has been identified as an important component in reducing stress and contributing to well-being during the perinatal period (Chasson et al., 2021), as well as in the context of infertility (Gourounti et al., 2012). A previous study found that women who reported higher levels of mastery at the beginning of MAR had lower perceived stress and greater life satisfaction (Ben Shlomo et al., 2017). A later study, conducted in MAR units in Israel during the COVID-19 pandemic, found that greater self-mastery was associated with lower levels of psychological distress among women in treatment (Ben-Kimhy et al., 2020).

The relationship between self-mastery and personal growth seems to be complex and inconsistent depending on the context. Previous studies have shown that a sense of mastery does not contribute to women’s experience of personal growth during childbirth and throughout the post-partum period (Sawyer & Ayers, 2009; Taubman – Ben-Ari et al., 2009), whereas a recent prospective study revealed that lower levels of self-mastery at early stages of pregnancy were related to greater personal growth in more advanced pregnancy stages (Mijalevich-Soker et al., 2024).

**Emotion regulation** refers to the process by which individuals manage their emotional experience in terms of intensity, duration, and expression (Gross & John, 2003). Two of the most well-documented strategies are cognitive reappraisal and expressive suppression (Gross, 2002; Preece et al., 2023). Through cognitive reappraisal, individuals change and reframe the meaning of a perceived situation and can thus reduce its emotional impact, while suppression involves inhibiting the outward expressions of one’s internal emotional state (Gross & John, 2003). The effectiveness of reappraisal has been well established, and it is often regarded as an adaptive strategy, helping individuals to manage negative emotions and promote positive feelings. In contrast, suppression is generally seen as a maladaptive form of emotion regulation, which accompanies greater negative affectivity and distress (Harp et al., 2024; John & Gross, 2004).

The association between these two regulation strategies and women’s well-being in the context of infertility has rarely been investigated. The existing evidence suggests that emotion regulation through reappraisal can alleviate the distress that arises in such circumstances (Ockhuijsen et al., 2014) and is linked to better quality of life (Domar et al., 2015). In contrast, emotional regulation through suppression does not contribute to infertility related distress, nor to anxiety and depressive symptoms (Chernoff et al., 2021; Hoyle et al., 2022).

The association between emotional regulation and personal growth has also rarely been studied, although the literature on personal growth in the perinatal context has gained momentum in the last decade (Brandao et al., 2020; Taubman – Ben-Ari et al., 2019). Taubman – Ben-Ari et al. (2022) conducted the study that is perhaps the most closely related this issue. The study assessed women during their early stage of pregnancy and examined the associations between their cognitive reappraisal and suppression, on the one hand, and personal growth, on the other. They found that whereas reappraisal was positively associated with personal growth, women’s emotional suppression was not related to their growth experience. Similar results were also found recently by K. Kim et al. (2023), in a study of personal growth among nursing students (mostly women) during the COVID-19 pandemic.

Alongside the internal resources of self-mastery and emotional regulation, **social sharing** has been found to be a significant contributor to both well-being and personal growth during challenging circumstances (Niederhoffer & Pennebaker, 2009; Slavin-Spenny et al., 2011; Tedeschi & Calhoun, 2004). However, when social sharing was studied in detail, the picture became less clear and mixed findings were presented regarding the contribution of sharing to positive outcomes. On the one hand, sharing negative emotions with others has been shown to relieve emotional burdens and reduce stress (Barasch, 2020; Slepian et al., 2017). On the other hand, such sharing can maintain and re-emphasize the difficulty, especially when the sharing continues for a long period (Curci & Rimé, 2012).

Studies focused on MAR specifically, have found that patients who share with others about their treatment experience less stress on a social and personal level (M. Kim et al., 2021; Martins et al., 2013). Furthermore, Peterson et al. (2025) found that individuals who more openly discuss their infertility with others report lower levels of depressive symptoms and experience greater meaning in life.

The ways in which individuals share their emotions also vary and, to a certain extent, depend on the affective content. Individuals tend to share with others on more distant platforms such as social media when it comes to positive experiences, whereas they are more likely to share on the phone or face-to-face when it comes to negative ones (Choi & Toma, 2014). To shed further light on the role of social sharing on women’s positive outcomes during MAR, we examined not only whether women share, but also to whom they confide and share their situation and to what extent, as sharing can have different implications depending on the social space in which the sharing occurs (Martins et al., 2013).

### 1.5. The Current Study

MAR consists of a prolonged stressful period that entails diverse challenges and adverse consequences both on a personal and social level (Peterson et al., 2025; Wells & Heinsch, 2020), which raises the potential for significant mental health problems (Benyamini et al., 2005; Skvirsky & Taubman – Ben-Ari, 2019). While most studies have focused primarily on the negative psychological consequences for women during MAR, several studies have raised the possibility of positive aspects that can be experienced during this period, such as well-being (Ben Shlomo et al., 2019; Swift et al., 2023) and personal growth (Abu-Sharkia et al., 2023; Paul et al., 2010).

However, knowledge about the contributors to these positive aspects is limited. Examining well-being alongside personal growth may be essential to providing a comprehensive picture of the positive aspects that can be experienced amid an extended period of uncertainty and stress. Identifying these outcomes in such challenging circumstances can provide valuable insights for health providers aiming to create focused intervention programs that improve patients’ mental health during MAR. Therefore, we examined the contribution of self-mastery, emotional regulation strategies, social sharing, and background and fertility-related characteristics to women’s well-being and personal growth during MAR. According to the theoretical model by Schaefer and Moos (1992), self-mastery and emotional regulation strategies represent women’s internal resources, whereas social sharing conveys environmental resources. Based on the literature review, the following three hypotheses and two research questions were formulated:

**H1.**  *Longer MAR period and more cycles will be associated with lower well-being and higher personal growth*.

**H2.**  *Higher self-mastery will be associated with higher well-being and personal growth*.

**H3.**  *A greater tendency to regulate emotions through expressive suppression will be related to lower well-being, whereas a greater inclination to regulate emotions by cognitive reappraisal will be associated with higher well-being and personal growth*.

(Q1) Is there a difference between the MAR groups (Ovulation induction, IUI, and IVF) in their experience of personal growth and well-being?

(Q2) Is social sharing related to well-being and personal growth?

(Q3) What are the unique and combined contributions of women’s background and treatment-related characteristics, self-mastery, emotional regulation strategies, and sharing with others to their well-being and personal growth during MAR?

## 2. Method

### 2.1. Participants and Procedure

The sample consisted of women aged 18–47 (*M* = 31.2, *SD* = 5.2) in the process of MAR to conceive their first child. Participants were recruited through a convenience sample through social media platforms. Following the university’s institutional review board approval of the study protocol (No. 012101), women provided their informed consent and completed several self-report questionnaires via the Qualtrics platform. The final sample consisted of 175 women, of whom 86% were married; 14% in a relationship; 84%; had an academic degree; and 16% completed secondary education; 47% defined their income as average; 40% above average; and 13% below average; 36% defined their health status as very good, 48% as good, 13% as fair, and below 3% as poor. Concerning MAR, 25.5% of the women underwent ovulation induction treatments, 25.5% intrauterine insemination (IUI), and 49% in vitro fertilization (IVF). The treatment period ranged from one month to 5 years (*M* = 1.2 years, *SD* = 0.95), and the number of treatment cycles ranged between 1–30 (*M* = 3.7, *SD* = 3.3).

### 2.2. Measures

**Posttraumatic Growth Inventory (PTGI; Tedeschi & Calhoun, 1996)** includes 21 items describing possible changes that can be experienced following a stressful event (e.g., “I established a new path for my life”). To measure women’s personal growth, the validated version adapted for the transition to parenthood (Taubman – Ben-Ari et al., 2011) and used in studies during the perinatal period in the context of infertility (Mijalevich-Soker et al., 2024) was utilized. Women were asked to rate to what extent they experienced each change since the treatment, marking their responses on a scale ranging from 0 (*I did not experience this change)* to 5 (*I experienced this change to a very great degree)*. A total score was calculated for each woman by averaging her responses to all items, with higher scores indicating a higher level of personal growth. The internal consistency was α = 0.93.

**Mental Health Inventory–short form (Stewart et al., 1988)** consists of five items derived from the original Mental Health Inventory (Veit & Ware, 1983), which measures an individual’s psychological well-being (e.g., “I felt happy”) and distress (e.g., “I felt sad and upset”) in the past week, with a response scale ranging from 1 (*never)* to 6 (*all the time)*. The total score for each woman was calculated by averaging her responses to all items, with higher scores demonstrating higher well-being. Cronbach’s alpha was α = 0.79.

**Self-Mastery Scale (Pearlin & Schooler, 1978)** consists of seven items designed to assess participants’ sense of control over their lives (e.g., “What happens to me in the future mostly depends on me”). Women were asked to rate their responses on a Likert scale, ranging from 1 (*strongly disagree)* to 4 (*strongly agree)*. The total score for each participant was calculated by averaging her responses to all items, with higher scores indicating a greater sense of self-mastery. The internal consistency was α = 0.80.

**Emotional Regulation Questionnaire (ERQ; Gross & John, 2003)** includes 10 items that measure an individual’s tendency to regulate emotions through cognitive reappraisal (e.g., “When I want to feel more positive emotion [such as joy or amusement], I change what I’m thinking about.”) and expressive suppression (e.g., “I keep my emotions to myself.”) Women responded to each item on a Likert scale from 1 (*strongly disagree)* to 7 (*strongly agree)*. The total score for each participant was calculated by averaging her responses to all items of each sub-scale, with higher scores indicating a higher tendency to regulate emotions using that strategy. Cronbach’s alpha for the reappraisal subscale was α = 0.84, and for suppression was α = 0.70.

**Sharing MAR with Others** consists of six items specifically designed for the current study, to understand the extent to which participants shared that they were undergoing MAR. Participants responded with a simple yes or no regarding whether they disclosed this information with various people, including their nuclear family, extended family, their partner’s nuclear family, friends, coworkers, and in online forums. Women’s responses were added to a single score reflecting the extent of their sharing with others, with a higher score indicating more sharing.

**A sociodemographic and infertility-related questionnaire** was used to obtain information on women’s background characteristics, including age, economic status (1 = below average; 2 = average; 3 = above average), education (1 = elementary; 2 = high school; 3 = post high school; 4 = academic degree), physical health (1 = poor; 2 = average; 3 = good; 4 = very good), marital status (1 = single; 2 = married; 3 = in a couple relationship without marriage; 4 = separated/divorced). Additionally, participants were asked to answer several questions regarding their MAR, such as the duration of treatment, the number of treatment cycles completed, and the type of treatment (1 = ovulation induction, 2 = intrauterine insemination (IUI), and 3 = in vitro fertilization (IVF)).

### 2.3. Data Analysis

Analyses were conducted using SPSS version 29 (IBM Corp., Armonk, NY, USA). First, a one-way analysis of covariance (ANCOVA) was conducted to examine statistical differences between the three types of treatment (ovulation induction, IUI, and IVF) in well-being and personal growth, controlling for women’s background characteristics (age, economic status, educational level, and physical health). Then, Pearson correlations were calculated to examine the associations between the study variables.

Next, two hierarchical regression models were performed to investigate the contribution of the study variables to women’s well-being and personal growth. In Step 1, background characteristics (age, economic status, educational level, and physical health) were entered. In Step 2, the types of MAR were added, specifically comparing IUI versus ovulation induction and IVF versus ovulation induction. For this examination, two dummy variables were created. The first variable was coded as IUI = 1 and ovulation induction = 0, while the second variable was coded as IVF = 1 and ovulation induction = 0. In Step 3, we entered the duration of treatment and the number of treatment cycles. In Step 4, we added self-mastery and the two sub-scales of emotional regulation (reappraisal and suppression) representing internal resources, and in Step 5, we entered the extent of sharing with others, as an external resource.

## 3. Results

Means and SDs of the study variables are presented in Table 1. The ANCOVA that examined differences in the dependent variables according to the three treatment types (controlling for background characteristics) showed no significant differences in well-being [F(2, 151) = 0.26, *p* = 0.76] and personal growth [F(2, 151) = 2.14, *p* = 0.12. The results of the Pearson correlations between the study variables also appear in Table 1.

Table 1 indicates that well-being was significantly and positively associated with self-mastery and cognitive reappraisal and negatively associated with the suppression dimension of emotional regulation. Personal growth was significantly and positively associated with self-mastery and the cognitive reappraisal dimension of emotion regulation and negatively associated with the extent of sharing with others. Lastly, there was no significant relationship between women’s well-being and personal growth.

The first hierarchical regression conducted to explore the contributions of the study variables to women’s well-being revealed that these variables accounted for 36.6% of the variance (Table 2). In Step 1, background characteristics explained 8% of the variance, with better physical health associated with higher well-being. Step 2 added only 0.3% to the explained variance, showing that the type of treatment did not contribute to women’s well-being. Step 3 contributed 7.6% to the explained variance, showing that the longer women underwent MAR, the lower their well-being. Step 4 added 20.7% to the explained variance, revealing that higher self-mastery and lower emotional suppression contributed to a greater well-being. Step 5 added social sharing, which did not contribute to the explained variance of well-being.

The results of the second hierarchical regression model accounted for 34.4% of the variance in women’s personal growth (Table 2). In Step 1, demographic characteristics explained 6.6% of the variance, with lower economic status significantly associated with higher personal growth. In Step 2, the type of treatment added 2.6% to the explained variance, revealing that women undergoing IVF reported significantly higher personal growth than women in the other types of treatment. Step 3 contributed an additional 2.4% to the explained variance, with significant contributions of longer treatment duration to higher experience of growth. In Step 4, women’s self-mastery and emotional regulation strategies contributed additional 20.5% to the variance, showing that a greater sense of mastery and a higher use of emotional regulation through cognitive reappraisal are linked to higher personal growth. Step 5 added 2.3% to the explained variance, indicating that the less women shared that they were undergoing MAR, the higher their personal growth.

## 4. Discussion

Contrary to the common tendency to focus on the negative consequences experienced by women undergoing MAR, we chose to direct our spotlight to positive aspects relying on Schaefer and Moos’s (1992) model and broaden current knowledge on the contribution of women’s background and fertility-related characteristics, self-mastery, emotional regulation, and the extent of their social sharing to their experiences of well-being and personal growth during MAR.

### 4.1. Associations Between the Study Variables and Women’s Well-Being

The findings revealed that among the background characteristics, physical health was the only background variable which was significantly associated with well-being and contributed to it in the regression model. It is not surprising that women with better physical health tend to report higher levels of well-being, as is well-documented in general (Hernandez et al., 2018). In particular, one study showed that women with better health during MAR are less stressed and are more satisfied with life. This study also indicated that they report higher levels of self-efficacy, which suggests that good health may contribute to a better ability to handle the physical challenges of the treatments (Ben Shlomo et al., 2017).

In addition, as expected, the shorter the treatment period was, the higher women’s well-being. This is also reasonable, as the longer the treatment duration, the greater the psychological, emotional, physical, and economic burdens (Cousineau & Domar, 2007; Sunkara et al., 2020), which may harm women’s well-being. The results also indicated that the number of treatment cycles a woman underwent was associated with well-being only in the simple correlative analysis, unlike the treatment duration, which contributed to well-being also beyond the consideration of other variables. This may indicate that the amount of time elapsed had much greater emotional weight than the number of cycles for women and impacts their well-being more significantly.

In line with our second hypothesis, we discovered that self-mastery and well-being were positively related, which was both validated through the correlation and regression analyses. The lack of control and uncertainty surrounding MAR can be one of the main sources of distress (Boivin et al., 2012; Palomba et al., 2018), so higher self-mastery may serve as a protective factor against the adversities resulting from the uncontrollable situation, which in turn manifests in higher psychological well-being.

Additionally, as expected, a higher tendency to suppress emotions was associated with a lower score of well-being, which is in line with the current literature (Fernandes & Tone, 2021). Regarding cognitive reappraisal, we surprisingly found that while other variables were considered, this strategy insignificantly contributed to women’s well-being. The expected positive association between these variables was found only through simple correlation analysis, which corresponds with previous findings about the benefit of this strategy (Harp et al., 2024; John & Gross, 2004). As adaptive emotional management is essential for patients’ mental health during such a difficult period, more research is needed to further understand the impact of these strategies in the context of MAR. Specifically, it is important to evaluate the effectiveness of cognitive reappraisal in helping women regulate their emotional states in this context.

### 4.2. Associations Between the Study Variables and Women’s Personal Growth

The study discovered that of all the sociodemographic variables examined, only lower economic status was associated significantly and contributed to greater personal growth. Our results join several other findings (e.g., Rozen et al., 2018; Taubman – Ben-Ari et al., 2024) showing that personal growth requires vulnerability. This also supports the basic claim of the posttraumatic growth theory (Tedeschi et al., 2018), which holds that growth arises out of stressful circumstances, which may include lower economic status. However, a recent study showed opposite results among Arab women undergoing MAR (Abu-Sharkia et al., 2023). Due to the limited and contradictory evidence on this subject, there is a definite need for further research to broaden understanding of the role of socio-demographic characteristics in women’s personal growth during MAR.

Our findings also indicated that women who underwent IVF experienced greater personal growth compared to the two other types of treatment (IUI and ovulation induction). This may correspond with a recent study which showed that women who conceived through IVF experienced higher growth compared to those who conceived spontaneously (Mijalevich-Soker et al., 2024). It is possible that because IVF is a more complex and invasive process than other medical procedures (Homburg, 2022), the decision to undergo this procedure requires more mental and tangible resources, and may be experienced by some women as a devastating experience that shatters their basic assumptions and parenthood aspirations (Schaefer & Moos, 1992; Tedeschi et al., 2018), making IVF more stressful than other MAR. Thus, this event may put women in more vulnerable and stress-evoking circumstances, increasing the likelihood of experiencing personal growth.

Further support for the idea that more challenging circumstances can foster personal growth may be evidenced in the finding that undergoing longer treatment was significantly related to and contributed to greater personal growth, consistent with the first hypothesis. Research on this topic remains limited and thus requires further exploration, not only due to the potential impact of treatment duration but also because our findings indicate that treatment duration may be more significant for women’s psychological outcomes than the number of treatment cycles. It is possible that a longer treatment duration indicates miscarriage, taking a break from treatment due to physical or mental health issues, or other challenges. These greater challenges may explain why it is the length of treatments and not the number of cycles that makes a significant contribution to personal growth. This explanation should be further explored in future studies.

In line with our second hypothesis, higher self-mastery was associated with greater personal growth and contributed to personal growth in the regression analysis. Interestingly, previous studies conducted during the perinatal period have reported different findings. For instance, in one study, lower self-mastery was related to higher personal growth during pregnancy (Mijalevich-Soker et al., 2024), while no relationship between these variables was observed in studies of the postpartum period (Sawyer & Ayers, 2009; Taubman – Ben-Ari et al., 2009). As far as we know, our findings are the first to show this particular relationship between self-mastery and personal growth during MAR.

To explain these mixed results of this and other studies, we considered the existing knowledge about the associations between the triangle of self-mastery, stress, and growth. Previous studies have indicated that moderate stress levels are associated with the highest level of personal growth; in contrast, very high or low stress levels reduce the likelihood of optimal conditions for growth among women (Rozen et al., 2018; Shakespeare-Finch & Lurie-Beck, 2014; Taubman – Ben-Ari et al., 2022). Additional studies have found that a greater sense of control during MAR and pregnancy is related to lower stress levels (Gourounti et al., 2012; Mijalevich-Soker et al., 2024). Taken together, it may be that greater self-mastery during a period of uncertainty, such as MAR, helps to moderate women’s stress levels, leading in turn to higher growth. It is possible that infertility is a unique period in the perinatal context, in which the sense of control over events is compromised more than during pregnancy and the postpartum period, when women may feel that they have achieved their desired results (conceiving or becoming mothers). Therefore, the role of self-mastery during pregnancy and post-partum is less significant for growth in comparison to the MAR period.

Concerning the association between emotional regulation strategies and personal growth, we found that, in line with our third hypothesis, using reappraisal to regulate emotions to a greater extent is related to and contributes to higher personal growth, similar to previous studies among pregnant women (Taubman – Ben-Ari et al., 2022). The tendency to reappraise the emotions raised by the situation seems to be consonant with processes that are known to foster growth, such as creating different meanings and developing a revised narrative around the challenging experiences (Jirek, 2017; Tedeschi et al., 2018). Thus, it is possible that women who initiated a process of reappraising the meaning of the situation were more likely to go through steps that foster growth. For example, considering that infertility can destabilize women’s internal worlds and threaten the coherence of their narratives, using emotional reappraisal may help them redefine internal narratives and adopt more positive perceptions of their stories, which can lead to personal growth.

The findings regarding social sharing indicated that women who shared their infertility with fewer people, whether at the family level, at work, or elsewhere, showed higher levels of personal growth, both in the correlation and regression analyses. Most of the literature on personal growth has focused on the benefits of self-disclosure (Lindstrom et al., 2013; Zhou et al., 2021), meaning sharing private information with others, not necessarily sharing specific content about events experienced (e.g., a disease diagnosis), but rather sharing emotional experiences or thoughts. Therefore, little is known about the relationship between sharing specific content and personal growth. Although the literature indicates that disclosing has positive outcomes in challenging situations, it is possible that in the context of infertility, which may involve external judgment or self-blame, the cost of sharing with others is greater than the benefit (Péloquin et al., 2018; Peterson et al., 2025). Thus, women who tend to keep their infertility to themselves may be less exposed to negative feedback both from themselves and others, and this may promote personal growth. Still, the inconsistency of our findings with other findings regarding the emotional advantages of sharing with others requires further investigation, specifically regarding women undergoing MAR (Barasch, 2020; Curci & Rimé, 2012).

### 4.3. An Integrative View

This study expands existing knowledge by showing the distinct roles of emotion regulation strategies in each of the measured positive outcomes. Among the two strategies, engaging in more cognitive reappraisal fosters growth, while lower levels of emotional suppression contribute to greater well-being. In addition, we propose self-mastery as a significant factor for the mental health of women undergoing MAR, as it contributes significantly to both well-being and personal growth. In contrast, social sharing may require further investigations as its effects are still unclear in this context, as we found no association between sharing and well-being, and that lower sharing with others about the fact they are undergoing MAR associated with more personal growth.

The study’s findings offer unique new perspectives on the topic of MAR, which is usually discussed and perceived in negative terms. It is undeniable that infertility and MAR entail many difficulties and complexities that take both mental and physical tolls, but this study presents a more multidimensional frame than has been offered so far on the subject. Our findings align with some claims of the dual continua model (Keyes, 2005; Tudor, 1996), which posits that psychological distress and well-being are interrelated yet distinct dimensions of mental health. In our case, whereas well-being represents lower distress, personal growth is a direct result of distress and can coexist alongside it. Our results emphasize the complex experiences women undergo during MAR, when positive aspects are experienced alongside the substantial challenges. Adopting this approach may be highly useful as it is suggested that focusing solely on psychological distress, without concurrently considering well-being or growth, provides an incomplete perspective on mental health and may limit clinical practice (Elkins, 2007; Kennedy et al., 2024).

To the best of our knowledge, this is the first investigation to examine both positive outcomes of well-being and personal growth simultaneously in the context of MAR, revealing several contributors and their distinct roles in each positive outcome. Furthermore, the results reinforce the distinction between well-being and personal growth, which has been mostly discussed in theoretical rather than empirical terms (Ruini et al., 2015; Taubman – Ben-Ari, 2012; Tedeschi et al., 2018). Further studies should focus on specific components that contribute to well-being and personal growth during MAR. This will enhance existing knowledge and may help to develop practical applications in clinical settings.

### 4.4. Limitations and Implications

Some limitations of the study include, first, a reliance on self-report measures, which may be subject to biases such as social desirability. Additionally, this study lacks medical information, which could provide a more comprehensive picture of the medical aspects related to women’s health and well-being during such a stressful period. Thirdly, our sample consists of Israeli women, a fact that may, to some extent, limit the generalizability of the results, as Israel has a very generous and unique medical system to address fertility issues. Fourthly, the study was conducted at a single point in time, which limits our ability to understand potential changes that may occur over the treatment period. Future longitudinal investigations may provide valuable insights into the trajectory of women’s well-being and personal growth over time. Lastly, we chose to concentrate on women who do not have children yet. A recent study indicates the importance of whether women in treatment already have children or not, finding that childless women exhibited lower levels of well-being compared to those who already have children (Bernet et al., 2025). Thus, future studies may wish to examine this characteristic in regard not only to well-being but also to personal growth.

It is important to address practical implications of our study findings, since psychological interventions during MAR can be beneficial in reducing distress and anxiety, and contribute to successful outcomes of MAR (Dube et al., 2023; Warne et al., 2023). We suggest that programs aimed at increasing patients’ self-mastery may have effective clinical implications and may help women go through this period with greater satisfaction and positive emotions, and lower stress levels, and contribute to their personal growth. Cognitive reappraisal may be a vital tool for women treated in fertility unit and could be offered by psychoeducational programs to help alleviate their distress during the treatment period. Taken together, we encourage fertility units and policymakers to enhance support systems and develop daily tools that help patients regain and increase their sense of control in these circumstances. This, in turn, may lead to better mental health outcomes and, hopefully, to improved conception results.

## Figures and Tables

**Table 1:** Pearson Correlations between the Study Variables.

	*Mean*	*SD*	1	2	3	4	5	6	7	8	9	10	11	12
1. Age	31.29	5.20		0.073	0.058	0.081	0.205 **	0.296 **	0.050	0.096	−0.020	−0.132	0.091	0.023
2. Education ^a^	4.08	0.77			0.268 **	0.069	−0.170 *	−0.081	−0.131	0.020	−0.161 *	0.070	−0.047	−0.072
3. Economic status ^a^	3.27	0.75				0.184 *	−0.115	−0.036	−0.011	0.014	−0.164 *	0.029	−0.063	−0.243 **
4. Health status ^a^	4.15	0.80					−0.104	−0.038	0.115	0.161 *	−0.206 **	−0.015	0.237 **	0.035
5. Duration of treatment (years)	1.25	0.95						0.622 **	−0.155	0.036	0.144	−0.246 **	−0.216 **	0.174 *
6. Number of cycles	3.76	3.39							−0.102	0.023	0.093	−0.185 *	−0.188 *	0.021
7. Self-mastery	2.74	0.49								0.345 **	−0.218 **	0.018	0.482 **	0.277 **
8. Cognitive reappraisal	4.62	1.09									−0.298 **	−0.014	0.238 **	0.419 **
9. Expressive suppression	2.84	1.21										0.163 *	−0.296 **	−0.142
10. Sharing with others	2.48	1.62											−0.021	−0.206 **
11. Well-being	3.44	0.78												0.088
12. Personal Growth	2.50	1.01												

Note: * *p* < 0.05, ** *p* < 0.01, ^a^ categorical scale between 1–5.

**Table 2:** Hierarchical Regression for Women’s Well-Being and Personal Growth.

		Well-Being				Personal Growth			
		*ß*	*t*	*p* Value	Δ*R2*	*ß*	*t*	*p* Value	Δ*R2*
Step 1					0.080 *				0.066 *
	Age	0.013	1.089	0.278		−0.003	−0.192	0.848	
	Education	−0.016	−0.194	0.846		−0.054	−0.495	0.621	
	Economic status	−0.118	−1.394	0.165		−0.322	−2.949	0.004	
	Health status	0.250	3.273	0.001		0.088	0.890	0.375	
Step 2					0.003				0.026
	IUI ^a^	0.114	0.655	0.513		0.210	0.946	0.346	
	IVF ^b^	0.017	0.106	0.916		0.412	2.050	0.042	
Step 3					0.076 **				0.024
	Duration of treatment	−0.180	−2.081	0.039		0.228	2.003	0.047	
	Number of cycles	−0.030	−1.298	0.196		−0.041	−1.358	0.176	
Step 4					0.207 ***				0.205 ***
	Self-mastery	0.596	5.200	<0.001		0.403	2.644	0.009	
	Cognitive reappraisal	0.020	0.369	0.713		0.307	4.323	<0.001	
	Expressive suppression	−0.121	−2.620	0.010		−0.047	−0.768	0.444	
Step 5					0.000				0.023 *
	Sharing with others	−0.006	−0.156	0.876		−0.105	−2.232	0.027	
	*R2*			0.366					0.344
	*F*(12, 156)			6.92 ***					6.28 ***

Note: * *p* < 0.05, ** *p* < 0.01, *** *p* < 0.001. ^a^ IUI = Intrauterine insemination, ^b^ IVF = In vitro fertilization.

## Data Availability

The datasets generated during the current study are available from the corresponding author upon reasonable request.
